# Cutting the Gordian knot: the blockage of the jejunal tube, a rare complication of Duodopa infusion treatment

**Published:** 2010-05-25

**Authors:** ML Negreanu, BO Popescu, RD Babiuc, A Ene, D Andronescu, RD Băjenaru

**Affiliations:** * Department of Gastroenterology, University Emergency Hospital, ‘Carol Davila’ University BucharestRomania; ** Department of Neurology, University Emergency Hospital, ‘Carol Davila’ University Bucharest Romania

**Keywords:** Duodopa, PEG gastrostomy, complications

## Abstract

We present the case of a 21–year–old man with advanced refractory Parkinson's disease treated with Duodopa continuous infusion. With this therapy, the patient had a spectacular recovery but after six months, he experienced an aggravation of his symptoms. A failure of his pumping system was suspected but we discovered that the jejunal tube was blocked due to a knot around a bezoar. This is the first complication of this kind described with the Duodopa infusion technique.

## Introduction

The continuous delivery of levodopa/carbidopa to the small intestine via a jejunal tube, formulated as a gel suspension (Duodopa), represents a new treatment method in patients with advanced Parkinson disease [1].  The continuous release results in less variability in levodopa concentrations and fewer motor fluctuations and dyskinesias than in oral administration [1].

This new technique implies a good cooperation between a neurologist and a gastroenterologist and uses a portable pump attached to a special tubing system. First, a classic PEG gastrostomy kit is placed under propofol sedation. This gastrostomy allows the passage of a pigtail catheter, which is deployed in the jejunum by using the endoscope and a rat tooth forceps. The procedure takes about 30 minutes in the expert hands and it can be carried out during a short admission in the hospital

The experience of European centers with this technique is increasing slowly mainly due to cost related issues. One of the largest series recently published included 65 patients followed for a medium period of 10.7 years [2]. Fifty–two patients were treated for more than 1 year. The adverse effect profile of levodopa/carbidopa infusion was comparable to that noticed after oral administration. Seven patients died, but all deaths were secondary to other causes and not to the procedure or the treatment. The most frequent problems were related to the intestinal tube placement, including dislocation of the stomach, which occurred in 69% of the patients during the first year [2]. The technical challenges posed by the enteral infusion system were offset by the improvement in motor fluctuations and dyskinesias offered by this technique for the majority of patients [2]. 

## Case presentation

A 21–year–old male patient with long standing severe Parkinson's disease was proposed for the Duodopa continuous infusion treatment. In June 2009, a PEG tube was inserted under general sedation with propofol and a jejunal tube was placed allowing the debut of the treatment. The patient had a spectacular evolution of his neurological disease. The only complication was pain at the insertion point, due to which he received symptomatic treatment.

**Figure 1:**
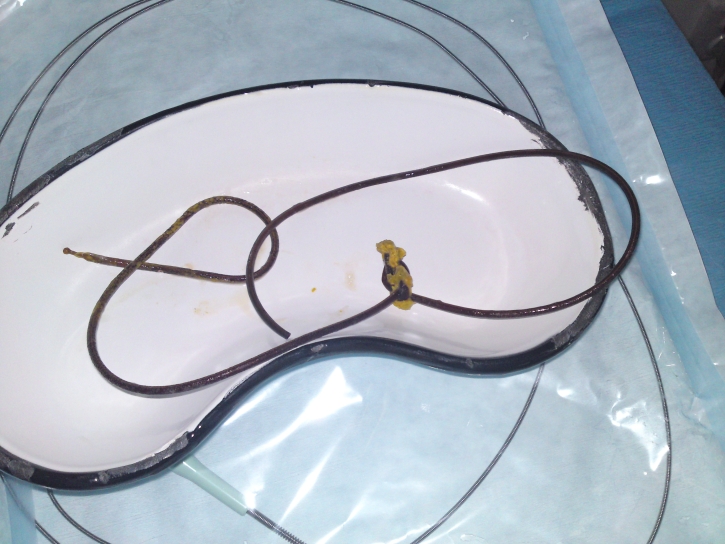
The jejunal tube after extraction

Approximately six months after the system placement the patient consulted his neurologist for a pump malfunction (high–pressure alarm; blockage) and the aggravation of his symptoms. After a check up of the infusion system, which worked fine, we decided to verify the patency of his tubing system. The tube clogging is a frequent complication after PEG placement [3] and this was our initial suspicion. An attempt to pass a guide wire over the jejunal tube showed in fact a blockage after 40 cm. The decision of tube removal was made and the patient had an upper endoscopy. The PEG gastric tube was at its place, in good position, but the jejunal tube was knotted in the stomach around a bezoar. (Figure 1)

**Figure 2:**
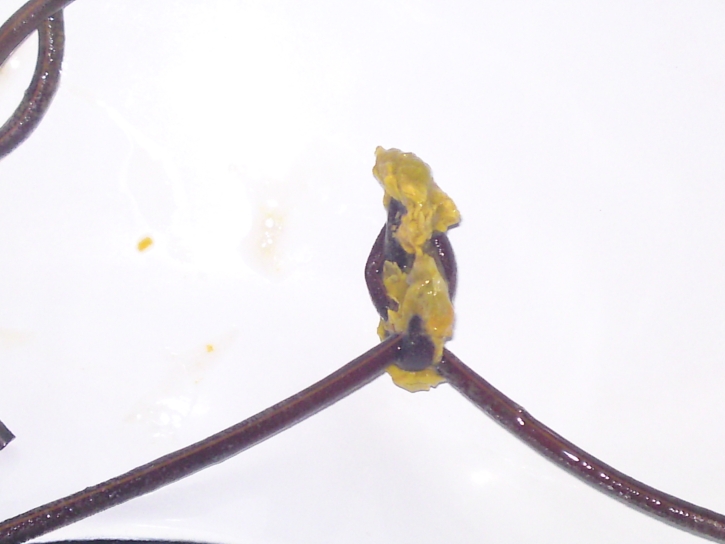
Jejunal tube, detail: A knot around a bezoar

We decided to extract it with a polipectomy snare after cutting its abdominal side. The extraction was made easily and without complications. (Figure 2)

**Figure 3:**
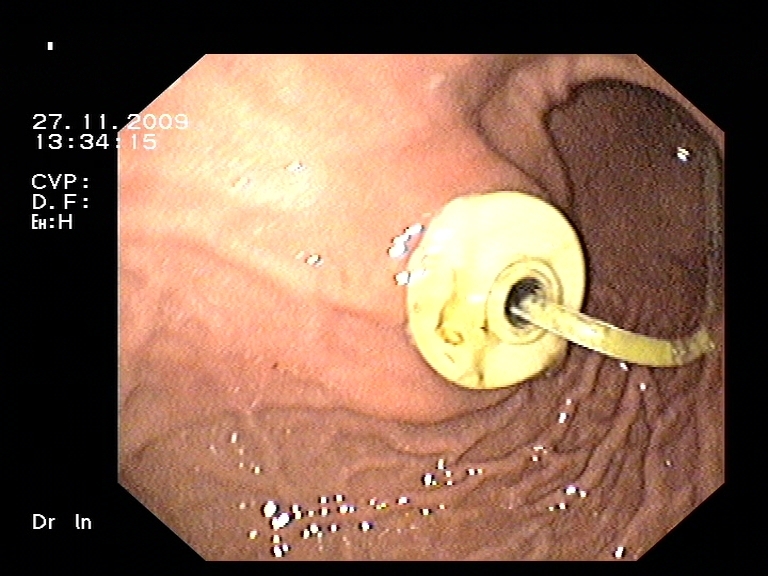
Endoscopic view of the replacement jejunal tube (in place over a PEG gastrostomy)

## Discussion

As far as we know, this is the first incident of this type, occurring with the Duodopa delivery system. The experience of seven cases (until now) in our centre is the biggest in Romania and showed no major drawbacks related to the delivery system montage or functioning. The PEG gastrostomy posing has a number of well–known complications but it is a common endoscopic procedure and, in expert hands, the complications are rarely seen [3]. 

With the Duodopa infusion method, no blockage due to a knot of the jejunal tube was yet reported. It came as a surprise to notice the way this knot was formed and, probably, the presence of the gastric bezoar and the patient's lack of care while manipulating its jejunal tube (normally a forbidden maneuver) may have played an important role. 
